# Patient Perspectives on Key Outcomes for Vocational Rehabilitation Interventions Following Traumatic Injury

**DOI:** 10.3390/ijerph18042035

**Published:** 2021-02-19

**Authors:** Kay Bridger, Blerina Kellezi, Denise Kendrick, Kate Radford, Stephen Timmons, Mike Rennoldson, Trevor Jones, Jade Kettlewell, on behalf of the ROWTATE Team

**Affiliations:** 1 Department of Psychology, Nottingham Trent University, Nottingham NG1 4FQ, UK; kay.bridger2017@my.ntu.ac.uk (K.B.); Mike.rennoldson@ntu.ac.uk (M.R.); 2 School of Medicine, University of Nottingham, Nottingham NG7 2UH, UK; denise.kendrick@nottingham.ac.uk (D.K.); kate.radford@nottingham.ac.uk (K.R.); jonesmob@ntlworld.com (T.J.); Jade.Kettlewell2@nottingham.ac.uk (J.K.); 3 Business School, University of Nottingham, Nottingham NG8 1BB, UK; Stephen.Timmons@nottingham.ac.uk

**Keywords:** traumatic injuries, return to work, vocational rehabilitation, outcomes, patient perspectives

## Abstract

Returning to work after traumatic injury can have a range of benefits, but there is currently little research that incorporates patient perspectives to identify outcomes of vocational rehabilitation interventions that are important to survivors. Trauma survivors (*n* = 17) participated in in-depth semi-structured interviews or focus groups exploring outcomes that were important to them for recovery and return to work. Data were analysed using thematic analysis. Participants identified a range of outcomes that they considered important and necessary to facilitate a successful and sustainable return to work: physical and psychological recovery, purposeful life engagement, managing expectations of recovery, managing expectations about return to work, and employers’ expectations. Our participants advocated for a multifaceted and biopsychosocial understanding of recovery and outcomes that need to be captured for vocational rehabilitation interventions. Implications for practice and research are discussed, and recommendations are given based on the findings.

## 1. Introduction

Injuries are a global public health concern, accounting for 9% of the number of deaths worldwide [1], and in 2013, an estimated 56 million hospital admissions [2]. Global costs of injuries and related illnesses range between 1.8 and 6.0% of GDPs across countries, with an average of 4% [3]. The injury mortality rate is decreasing over time in most countries [4]. As survival rates increase, other injury outcomes increase in importance. Injuries result in substantial disability, accounting in 2013 for 10% of disability-adjusted life years [2] globally. Problems with physical [5], psychological [6], social [7], and occupational functioning [8], pain [9] reduced quality of life [10], and fatigue [11] are common post-injury, with many trauma survivors experiencing long-term disability [12].

Health systems across the world have developed in various ways to respond to such issues. For example, in England, services for treating injured survivors were reconfigured in 2012 with the establishment of Major Trauma Centres (MTCs). These MTCs provide [13] care for survivors with at least moderately severe injuries (Injury severity score (ISS) ≥ 9), which are most commonly caused by falls, road traffic collisions, assaults, and penetrating injuries [14,15] and currently report survival rates of over 90% [15].

Return to work is a problem for many injury survivors [16], with approximately one-third of those admitted to hospital not returning to work within twelve months post-injury [17]. A review of studies following traumatic brain injury (TBI) survivors found that only 41% [range 0–85%] were employed 12–24 months later [18]. Another review found that among those with mild TBI, the proportion of those who had returned to work at 6 months was more than 80% [19]. People with TBI who do not return to work within 2 years are unlikely to work again [20]. Return to education following traumatic injury is less well documented. An Australian study of young adults following TBI showed that only 12% were still studying and 29% were employed after the injury [21]. Work participation has benefits for physical and psychological health, quality of life, financial wellbeing, connection with others [22], positive identity, and status [23]. 

A range of vocational rehabilitation (VR) interventions have been developed to support return to work post-injury [24,25,26]. However, it is those interventions that are informed by biopsychosocial models of health and rehabilitation that offer the most comprehensive integration of factors influencing readiness to return to work after injury [27] given that barriers to work are multifactorial [26,28]. For example, Loisel and colleagues argue that successful interventions should aim to address multiple factors that can contribute to disability prevention: the characteristics of the individual and the workplace, as well as accounting for the wider system of healthcare and welfare. Similar to Loisel and colleagues’ work, the International Classification of Function Disability and Health (ICF) [29] advocates for a biopsychosocial framework to understand the different factors that contribute to workplace disability. A systematic review of health outcomes after major trauma concluded that existing outcome measures do not fully describe the impact of major trauma on function, disability, and health. Measuring of health outcomes for trauma survivors may be inaccurate or developed for other populations [30], and outcomes measured in intervention studies may not translate into clinical benefits for survivors [31,32]. In fact, systematic reviews of VR studies show that outcome measures tend to be chosen for ease of statistical comparison (e.g., time taken to return to work [28]) or secondary measures such as functional status, pain or mental health [33], potentially not capturing more complex contributors to return to work [34]. 

As trauma survivors form a heterogeneous population, there are a large number of potential outcomes to measure in VR intervention studies. Existing systematic reviews of return to work following various types of injury identified a range of outcomes such as return to work, sick leave/absenteeism, duration of work disability outcomes, disability compensation status, job loss, work participation/functioning, activity limitations, and participation restriction [35,36]. On the other hand, these reviews noted heterogeneous factors as predictive of successful return to work. The strongest evidence is for social and psychological predictors including level of education [33,35] occupation type [36], hand occupation type [36], and self-efficacy [35]. Biological predictors of return to work include pain, severity of injury [35], and functional status [33], but injury severity may be inconsistently defined [33] and not comparable across studies. Age and gender were also found to be inconsistent predictors [33]. Such range of predictors supports a biopsychosocial approach to return to work. A further issue in the current understanding of outcomes is that it is not clear which outcomes are important to trauma survivors in relation to return to work or education. Outcome measures need to be relevant, appropriate, represent the priorities of those with lived experience of injury, and address the information needs of other stakeholders (e.g., service providers, employers, commissioners, policy makers), and society [31,37]. There is evidence to suggest that survivors’ perspective impacts what constitutes successful return to work [34] and recovery from injury [38]. Therefore, the aim of this study was to explore lived experience perspectives on outcomes important to trauma survivors specifically in terms of returning to work or education. 

## 2. Materials and Methods 

### 2.1. Participants and Materials 

Semi-structured interviews or focus groups were conducted with trauma survivors to allow in-depth exploration of the topic. Participants were initially invited to participate in focus groups but where these were not preferred or a shared timing could not be agreed, participants took part in interviews instead. Offering different formats allowed participants to make their preferred choice as well as enabling the use of different methodological approaches, which have different strengths. [39]. For example, focus groups enable participants to compare their views with peers, while interviews enable more in-depth information about personal experiences [40]. Seventeen trauma survivors from three UK locations (Nottingham, London, Leeds), with a range of injuries were interviewed (*n* = 10) or took part in focus groups (*n* = 7 over 3 groups). Trauma participants not recruited in hospital settings are a hard to reach population, so we advertised the study widely to all existing study team rehabilitation contacts and through trauma charities (After Trauma, London; Day One, Leeds) as well as social media and included all those that expressed interest in taking part in the study. The researchers travelled to these locations to facilitate face-to-face participation where possible. Participants were included if they were aged ≥18 years, admitted to hospital for ≥3 days after injury, working or studying at the time of injury, and able to provide informed consent. The study aimed to recruit participants from a range of employment backgrounds, socio-demographic backgrounds, and types of injuries, and it extended the recruitment time to ensure the participation of under-represented groups such as BAME (Black, Asian and Minority ethnic). Most participants had returned to work following their injury (14/17, 82%); however, only three participants (18%) had received VR support. This VR support did not constitute a formalised intervention (rarely provided in the UK at time of research); instead, it refers to work-focussed support from a rehabilitation professional (occupational therapist *n* = 2 or charitable support worker *n* = 1), which included workplace liaison. Neither of the student participants received this. A summary of participant characteristics is shown in Table 1. Interviews and focus groups were conducted by two authors (JK and KB) and a Masters student. Most interviews were conducted by telephone (*n* = 8) to enable the inclusion of participants whose injuries were a barrier to travel. Focus groups were conducted in person but were smaller than planned. However, the smaller size allowed participants to focus on comparisons in their experiences of injury and recovery and how this affected return to work. One focus group included two participants who volunteered in a peer support charity, and the small focus group size allowed valuable reflection on their wider experience as trauma survivors. The decision to stop recruitment at this number was influenced by the fact that no new themes were identified in the final transcripts that were reviewed. 

The interview and focus group topic guide was informed by PPI (Public and Patient Involvement) members of the research team and existing research on outcome measures [30,41]. The topic guide enquired about life after injury, impact of injury on physical and psychological wellbeing, ability to work, post-injury goals, vocational and psychological support, outcomes important to participants for recovery and return to work, and views on the goals that VR interventions should aim to achieve. Ethical approval was obtained from the University of Nottingham Faculty of Medicine and Health Sciences Research Ethics Committee (Ref: FMHS 150-1811) and Leicester South NHS Research Ethics Committee. Recruitment took place from 1 February 2019 to 31 January 2020. The Consolidated Criteria for Reporting Qualitative research (COREQ) checklist [42] was used to report methodology and analysis. 

### 2.2. Analysis 

Interviews and focus groups were audio-recorded and then transcribed verbatim. Data were analysed thematically using Braun and Clarke’s (2013) [43] analytic steps within NVivo software (QSR International, US). This type of analysis was used because it allowed the identification of key patterns across participants with diverse experiences and backgrounds. Data were coded by one author (KB) after multiple reading of transcripts. The coding frame and the detailed coding of one transcript was discussed in detail with two other authors (BK, MR) at the start, middle, and end of the coding. Coding focussed on the full transcripts where reference was being made indirectly to outcomes (e.g., Tell me about your return to work journey?) as well as explicit responses to questions addressing goals, outcomes, or issues important to trauma survivor recovery. Codes were categorised and organised into themes and subthemes, which were discussed and agreed with five authors (KB, JK, KR, BK, MR) as well as the PPI team and wider research team. Several steps were undertaken to ensure the themes were independent, coherent, and accurate. A summary of the core narrative and essence of each theme/subtheme was written. Next, a map of all themes and subthemes was created and discussed among the research team. Finally, the transcripts were revisited to ensure the accounts were coherent and represented the dataset accurately. Where disagreements arose, analyses were discussed until consensus was reached, and the final analysis structure and write up was led by a different author to the one who coded the data (BK). A PPI member of the team took part in the discussion of the different themes and provided detailed comments on the validity of the core interpretations and patterns identified against their lived experience as well as contributing to the writing process. The main findings are illustrated with participant extracts, followed by information on the participant’s injury, gender, and age. 

## 3. Results 

Participants all had lived experience of traumatic injury and were invited to contribute their perspectives on the outcomes that were important to them in their journey back to work or education. They identified a range of outcomes they considered important and necessary to facilitate a successful and sustainable return to work. Three main themes (Table 2) were identified: (1) physical and psychological recovery and purposeful life engagement; (2) managing expectations of recovery; and (3) managing expectations about return to work and employers’ expectations. The number of participants in each category did not allow in-depth comparisons to be made between the accounts of those participating in the focus groups or individual interviews. 

### 3.1. Theme 1: Physical and Psychological Recovery and Purposeful Life Engagement

Physical, psychological recovery, and purposeful life engagement were all important interrelated outcomes that influenced return to work. Subtheme 1 focusses on physical needs, subtheme focusses 2 on psychological needs and subtheme 3 focusses on purposeful life engagement. 

#### 3.1.1. Physical Recovery

Participants spoke about their primary goal being physical healing, particularly where there was significant uncertainty about recovery time. For some people, thinking about return to work was influenced by their physical limitations and the need for surgery, physiotherapy, and general healing, and physical recovery was a necessity before other activities, such as return to work could take place:

*“…my brain wasn’t really processing what had actually happened because I was so concentrating on like right, you’ve got to learn to walk again” (amputation, female, twenties)*

*“…I had goals that the physio team had put me and I wanted to break the goals. I wanted to beat them a week earlier, a day earlier. And I went for it really…” (polytrauma, male, forties)*

This prioritisation of physical recovery is not surprising, although for many participants, recovery was not purely physical. 

#### 3.1.2. Psychological Recovery

Psychological health was an important part of recovery, which impacted on return to work:

*“It was a very difficult period of time, you get—mentally it’s difficult to get through it, never mind the physical and what you’re going through, mentally it’s a difficult time.” (pelvic fracture, female, fifties)*

Other psychological issues that arose included the negative impact of returning to work too soon:

*“My brain went from doing nothing to being a 100 mph in the space of a morning. It was just like, oh… it just adds to a level of stress that I didn’t need.” (lower limb, male, thirties)*

*“I felt I was a prisoner. I got up in the morning, I was a prisoner in the house.” (lower limb fracture, female, sixties)*

Some participants had needed psychological support to help identify and address issues, such as isolation and anxiety and preparing for return to work.

*“I think through the psychological support sussing out what the most pressing issue is, what’s causing the anxiety, and then trying to find a service or a network of people.” (spinal cord injury, female, forties)*

*“Seek out clinical psychology. I’d make that your number-one goal without a shadow of a doubt… I’d say, don’t go back until you’re mentally ready for it. I think that’s the main thing.” (polytrauma, male, forties)*

For some participants, psychological support was essential to prepare for return to work. Overall, participants highlighted different ways the injury impacted them psychologically (e.g., depression, anxiety, post-traumatic stress, loneliness), which suggests the importance of measuring a range of psychological outcomes of rehabilitative interventions.

*“Anxious first, and that made me isolate myself, and then the isolation caused the depression. I can see that as a pattern.” (spinal cord injury, female, forties)*

*“When it comes to the anniversary of the accident, (…) You know, you have weird flashbacks.” (amputation, female, twenties)*

Thus, psychological recovery for a range of psychological outcomes was an essential part of preparation for return to work, and for some participants, this could only be achieved by receiving appropriate support. 

#### 3.1.3. Purposeful Life Engagement as Part of Recovery

Some participants spoke about need to regain a sense of purpose in life, and for some, this was part of their motivation to return to work. This was especially important where return to prior employment was not seen as feasible or desirable:

*“I think it’s important to try to get back to work if you can, because it clearly gives you a sense of purpose in your life, which you may feel yourself to be useless… I think it is important to feel you have some use, not just to yourself but to those in society.” (spinal cord injury, male, sixties)*

*“But still there’s some steps towards getting a worthwhile, feeling satisfied with yourself and that you’re fulfilled.” (polytrauma including traumatic brain injury, male, forties)*

For others, a less demanding interim role or volunteering opportunity provided them with purposeful activity that bolstered their sense of recovery.

*“…Once I got reasonably comfortable with myself, I started off my endeavour back to work, it wasn’t a planned thing, but I wanted to give myself something to do, I was, a purpose in life as people say, and it started off for me volunteering.” (polytrauma including traumatic brain injury, male, forties)*

*“I’m worrying that I’m not smart enough for this but I feel I need a job even for independence and becoming a normal person again… there was just a coffee shop near my mum and my dad and there was a sign, looking for staff, so I went in.” (polytrauma, female, thirties)*

An important part of recovery for some of the participants was engagement with work-related activities, which could contribute to a sense of purpose and meaning in life. In turn, this engagement contributed to recovery.

### 3.2. Theme 2: Understanding of Normality and Managing Expectations 

Participants viewed returning to normal as an important outcome (subtheme 1), along with understanding recovery timelines and managing recovery expectations (subtheme 2).

#### 3.2.1. Returning to Normal 

Participants expressed a strong desire to return to pre-injury life, including pre-injury routines, independence, and return to work.

*“I’m very driven by my work, so for me, it was about finding normality again…I’ve heard a lot about the importance of routine and when you’re back into your normal routine, how that can kind of benefit you psychologically.” (amputation, female, twenties)*

Part of returning to normal was achieving independence and being able to undertake daily tasks. Participants expressed a desire to cease being dependent on partners or family members and frustration with physical impairments (e.g., being stuck at home or unable to perform basic self-care tasks):

*“…The difficult thing is… you’re dependent on everybody when you did everything for yourself.” (lower limb fracture, female, sixties)*

*“So, getting all of it back and feeling like I was back to being independent, yeah.” (lower limb fracture, female, thirties)*

For others, returning to normal equated to resuming activities such as exercise, which was seen as a precursor to being able to return to work.

*“…I knew once I was able to start going swimming and doing a bit more bike riding, I would be all right to stand at work” (lower limb fracture, female, sixties)*

One participant noted that returning to work too soon had resulted in her becoming more dependent at home (due to fatigue) impacting on work/life balance for the household. This type of experience highlights the inter-connectedness of outcomes:

*“…You could, of course, go back to work before you start doing things like walking the dog, because working is essential and walking the dog is non-essential. But for me, independence did involve that because otherwise, I was putting this burden on my partner and frustratingly, I think, returning to work meant that it was longer before I could start doing things like walking the dog because if I worked on Tuesday I was ruined on a Wednesday.” (lower limb fracture, female, thirties)*

Returning to normal was an important outcome, and return to work was part of this returning to normal. However, returning to work had to be balanced carefully with functioning in other important aspects of life, highlighting again the complexity of recovery and health and wellbeing. 

#### 3.2.2. Managing Recovery Expectations 

Recovery expectations were viewed as an important outcome. Many participants found that not knowing the expected timeframe for physical and psychological recovery was problematic in terms of psychological adjustment and managing emotions.

*“I wasn’t very patient. I wanted to not feel anxious in the morning, to not feel depressed in the morning, to have no further bladder infections in the morning. So, I wanted everything to be fixed, and it doesn’t work like that.” (spinal cord injury, female, forties)*

Where participants had received more precise medical estimates regarding expected recovery, they reported greater wellbeing than where there was greater uncertainty.

*“She [nurse] was brilliant. She told me, and it did happen like that. I was like, ‘All right then.’ This, this and this, and this could happen. Well it did happen, so I thought that’s really, really good.” (pelvic fracture, female, fifties)*

An important part of recovery was understanding and managing the impact of the injury and the recovery process. Health providers had a crucial role in facilitating this understanding, which when present was perceived to be beneficial. 

### 3.3. Theme 3. Managing Return to Work and Employer Expectations 

Participant (subtheme 1) and employer expectations about return to work, employer awareness of the impact of injury (subtheme 2), support for employees, and handling work-related goals and tasks (subtheme 3) were all important outcomes.

#### 3.3.1. Managing Participants’ Return to Work Expectations 

Managing expectations was seen as a beneficial part of planning and negotiating return to work. Participants referred to finding a new work normal and recovering to a stage that was not necessarily a return to the pre-injury state.

*“If the employer is in a position to keep a job open, to just give them an outline of, maybe, timescales. You know, they’re not off sick because they want to be off sick. Whatever the trauma is, whatever the disability is, it’s a major life-changing event. It’s not just a case of, right, she or he is out of hospital, so therefore, why aren’t they back at work?” (spinal cord injury, female, forties)*

*“I think for lots of people it is, ‘I need to get back to work’. Sometimes it’s the first or second thing they might say, how long did it take you? We’re all different, but I need to get back to work.” (pelvic fracture, female, fifties)*

Participants recognised they had (or had not) been supported towards making realistic decisions that would result in a successful and sustainable return to work. Some had underestimated the mental fatigue and stress of the change from convalescence to full-time work. 

Those who had been guided to undertake a phased return by an occupational therapist had not recognised how important that would be. This applied to people with a range of injuries.

*“…As long as you’re prepared to go back to exactly how you were before, and I don’t think I was. I think I should have taken on some reduced duties or something first. That was my choice. They offered that and I said I’d be fine and then it turned out pretty bad for me.” (lower limb, male, thirties)*

#### 3.3.2. Workplace Understanding Impact of Injury, Including Invisible Impacts

Participants emphasised the importance of their workplace understanding the nature of their injury and its impact on their work. This was particularly true for less visible impacts, such as memory, attention span, fatigue, pain, medication side effects, Post traumatic stress disorder (PTSD), and incontinence.

*“…Especially for newly injured people, I think that your team leader, and maybe a few people on the team that you work in, should be educated a little bit about spinal cord injury, because then there are people, even if it’s not the whole team, looking out for you, looking out for those signs. Maybe you could say, right, there are three people that you can just say, ‘I’m really struggling today,’ …liaising with the employers, potentially liaising with chosen colleagues—because you don’t want everyone knowing your business, but a select few people that can sort of look out for you.” (spinal cord injury, female, forties)*

Participants explained that workplace understanding could also be beneficial as it facilitated employers’ interactions to meet their needs in a timely manner.

*“…Having someone coming to see you at the workplace, and having a chat to your manager about what you might need…I had a really good experience in terms of my management, I do know of some organisations in where the line managers are new, fresh to post, wanting to make a difference, and actually see absence from work as a black mark against them, as not motivated, rather than thinking about the individual’s needs.” (upper limb fracture, female, sixties)*

The workplace understanding of the nature of the injury and its impact on the individual and their ability to work was considered an important outcome. Thus, employer and co-worker awareness of the impact of injuries could be beneficial for a more positive experience of return to work and for the provision of needed support or adaptations.

#### 3.3.3. Successfully Managing Individual Expectations and Options Available 

Developing realistic expectations about return to work appears to be facilitated by using patient-focussed goals, tailored to the needs of the patient, which help maintain a sense of hope and achievement. These findings suggest that meaningful and personalised goals are important outcomes post injury.

*“It’s like getting somebody into work, it should be personal to them, it should be focussed on what their goals are, how soon they want to get back, and what getting back to work looks like for them… Some people might see getting back on a phased return within three months a success, and other people like me will see getting back as soon as possible full-time with as little disruption to your life as a success… I think make it patient-focussed and understanding what the needs of each individual are and tailoring it to them.” (lower limb fracture, male, thirties)*

Other participants spoke about the importance of goals that are broken into small, achievable steps that help maintain a sense of progress, for example volunteering if returning to pre-injury employment is not possible.

*“So meaningful baby steps that, because you know, going back to work in your old job feels a million miles off, you just can’t do that, there’s no way, I’m never going to be asked. Whereas if I can set some goals that are achievable, you know baby steps is the way as far as I’m concerned.” (polytrauma including traumatic brain injury, male, forties)*

The process of recovery, where goals are personalised and achievable, was an important part of recovery and should be captured as part of outcomes. Overall, the data highlighted the multifactorial and inter-related nature of outcomes important to trauma survivors as they negotiate return to work. 

## 4. Discussion

### 4.1. Main Findings 

This study explored outcomes that trauma survivors felt were most pertinent to their recovery and pathways back to work or education. At the core of the findings is the multifaceted and biopsychosocial understanding of recovery, which includes physical health, psychological health, purposeful engagement and management of expectations for self and employers, and recovery process. The findings also suggest the connection of the different aspects of recovery whereby earlier steps (physical, psychological, and purposeful engagement) impact on the later ones in relation to return to work. However, these connections do not just flow in one direction, as the different aspects of recovery are interrelated. For example, uncertainty is a cross-cutting issue across the different aspects of recovery presented in all the three themes. These personally important outcomes were unlikely to be fully captured by those routinely measured in VR interventions [28,33], particularly where they relate to rehabilitation processes. Prior research argues for the importance of outcomes that capture the complexity of factors outlined in the ICF domains [33]. The present study does capture this complexity and brings a novel contribution to the understanding of recovery and pathways to return to work and education by identifying a range of complex outcomes and the ways they related to each other. 

### 4.2. Comparison to Prior Research and Theory 

Both physical and psychological recovery, including a range of mental health issues (e.g., anxiety, depression, isolation) were mentioned by the participants as important outcomes. This very much reflects existing evidence on the impact of injuries, which includes both physical and psychological outcomes [44,45,46,47] and the multiple factors reflected in biopsychosocial vocational rehabilitation theories [27]. However, an important finding in this study is that trauma survivors also expressed the need for having a sense of purpose or purposeful engagement, supporting a recent qualitative exploration of return to work following serious injury [48]. This may be a response to traumatic events triggering changes in the meaning of life for survivors [49] such as learning to appreciate different aspects of life. The focus on meaning of life is at the center of post-traumatic growth theories of trauma [50] and psychological wellbeing theories [50], but it is not clearly acknowledged in vocational rehabilitation theories [51,52].

Many participants talked about returning to normal, regaining independence, and being able to undertake daily tasks themselves. Returning to work was seen as a step towards independence and normality. This finding supports previous research, particularly the desire to return to normal life following traumatic brain injury (TBI) [53] and highlights the significance work has on an individual’s identity and long-term recovery [54]. In the TBI literature, return to work is considered an important aim of the rehabilitation process, so interventions use it as a measure of successful recovery [55,56]. However, for the participants in our study, return to work had to be managed carefully, as returning too early could have negative effects on employment and recovery, again supporting a previous qualitative study [48] noting the importance of balancing return to work against other needs. This reflects existing work with TBI survivors who require continuous support, given the ongoing challenges they face due to fatigue and memory problems [57]. These findings also highlight the importance of taking a biopsychosocial approach to understanding competing outcomes, whereby return to work as well as physical and psychological recovery need to be addressed. This reflects previous research with serious injury survivors, which concluded that successful return to work depends on addressing both personal and contextual needs [48].

Managing expectations was important to participants, in terms of timelines for recovery and return to work. Uncertainty around timelines was unsettling for participants. In addition, some participants lacked insight into how severe their injury was and/or how it might affect their ability to work, supporting prior research in the area [58,59]. Management of return to work was closely linked to management of expectations about recovery as well as management of work expectations following injury (ability to return to work) and employer expectations (impact of injury on work). These findings support past research, which has shown that expectation management about recovery can influence a range of outcomes such as functional outcomes and pain [60] as well as influence expectations about return to work [61]. Other options needed to be considered when available (part-time or volunteer work). Again, these findings reflect the biopsychosocial approach by highlighting the multiple outcomes that need to be addressed by VR intervention. For example, Loisel and colleagues [27] refer to the importance of patient reassurance, while ICF [29] recognises the importance of family and workplace support in return to work. 

In terms of outcomes measures for VR interventions, it is likely that some of the issues raised around complex recovery, expectation management, and preparation for return to work have not been fully captured in current research [62]. The outcomes outlined by survivors highlight the importance of individual experiences and needs as well as a personalised understanding of recovery. Past research has shown the importance of personalised understanding of recovery [38,63] that goes beyond physical functioning. Defining personalised outcomes can be difficult, but not doing so risks ignoring what is most important to the patient [64,65]. For example, returning to work might be important to a patient, but the process of returning to normal or providing for their family, reducing work hours, withdrawal from work, or better quality of life might be the goal they are working towards. This multiple-need approach supports the biopsychosocial model that underpins rehabilitation and patient-centred care, and it can improve outcomes [66,67]. Complex interventions such as VR should focus on understanding an individual’s personal, social, and physical context, as well as work and wider health and welfare context (e.g., presence of specialist VR) and what is important to them following trauma (i.e., their goals and purpose). 

VR interventions need to carefully balance measurable outcomes that are important for service providers and commissioners of services, while acknowledging that other outcomes and processes are important for individuals with lived experiences of illness or injury. This study showed that a range of physical, psychological, social, and employer outcomes is needed. In addition, it highlights how the process of rehabilitation can be as important to recovering for trauma survivors as the outcome [68]. This finding is corroborated by other healthcare research advocating for the inclusion of specific process measures to complement outcome measures of interventions [69] as well as survivors’ own perspectives in defining quality of care [59]. This also reflects past work with injured survivors that highlights the importance of the process of care in satisfaction with the care [59]. Process as well as outcome should also be integrated in the measurement of outcomes from VR interventions. 

### 4.3. Strengths and Limitations 

This study explored outcomes important to individuals following hospital admission for a diverse range of traumatic injuries, specifically in terms of returning to work and VR interventions. To the authors’ knowledge, no other studies have investigated these issues in depth in such a wide range of survivors, which includes BAME populations (e.g., Black, Asian and Minority ethnic). The diversity of the sample, while useful, highlights the need for investigating the experiences of the different populations in more depth to identify any differences in VR needs. It was not possible to explore the impact of VR interventions received by participants following their injury on their experiences of return to work or education. This is due to the limited number of participants receiving such specific support and the informal nature of the support received. Future qualitative research should explore this further. In addition, the time since injury ranged from 6 months to 14 years as we aimed to understand both shorter- and longer-term processes and outcomes. It is possible that those who had experienced the injury recently were still undergoing recovery and reflected more on shorter-term outcomes, while those who had the injury many years prior to the interview reported longer-term outcomes. It is also possible that recall of experiences from over a decade ago could be poorer due to the time lapse or even be affected by later life events. 

The use of semi-structured interviews and focus groups enabled participants to discuss outcomes that were important to them, the reasons why they were important, and how they relate to return to work. The research team included expertise from a range of academics and practitioners who specialise in VR, psychological support, trauma, and health services research as well as PPI members, which enabled a multi-perspective interpretation of the data. 

### 4.4. Implications for Research 

Future, larger studies would enable an identification of outcomes for different traumatic injuries and variation in priorities between different injury groups. Future research should also investigate the role of other patient characteristics (e.g., socio-economic status, type of employment/education, and caring responsibilities), which might influence recovery and the process of return to work or education. 

### 4.5. Implications for Practice 

The design of future VR interventions for trauma survivors should include measuring a wider range of processes and outcomes than often previously used. These include physical and psychological wellbeing, recovery and work expectations, purpose in life, employer awareness of the impact of injury, and achievement of personalised goals [70]. 

## 5. Conclusions 

Returning to work after injury is an important marker of recovery and a frequently used metric for measuring the effectiveness of rehabilitation interventions and health service outcomes. The range and complex nature of injuries among individuals presenting to major trauma services, the multi-component interventions needed to address them, and the individualised nature and meaning of recovery to the injured person require rehabilitation services to capture a range of outcomes and processes that are linked to the individual’s goals and sufficiently sensitive to capture individually tailored interventions. These must go beyond measures of function and take account of meaningful activities and life roles. They should also account for anticipated variation due to injury type, the rehabilitation approach, and psychosocial and environmental factors, which might influence recovery and return to work. The process of returning to work/education and recovering (e.g., regaining a sense of purpose) can also be perceived to be as important as the other outcomes, something which is difficult to define in terms of measurable trial outcomes. Our findings support the biopsychosocial model, which is at the core of rehabilitation interventions and underpins patient-centred care. Our study also highlights the value in involving people with lived experience in identifying the most relevant outcomes for specialised interventions like VR.

## Figures and Tables

**Table 1:** Summary of trauma survivor participant characteristics.

Participant Characteristic (*n* = 17)	Number/Range
Age	27–68 years (mean 44)
Gender	Female (*n* = 10); Male (*n* = 7)
Injury type	Amputation *n* = 1 Lower limb injury *n* = 5 Pelvic injury *n* = 2 Polytrauma *n* = 3 Polytrauma + TBI *n* = 1 Spinal cord injury *n* = 2 TBI *n* = 2 - Upper limb injury *n* = 1
Time since injury	6 months to 14 years
Ethnicity	White British (*n* = 15); Asian (*n* = 1); Black British (*n* = 1)
Employed status at time of injury	Employed (*n* = 11); Self-employed (*n* = 4); Student (*n* = 2)
Pre-injury employment type/sector	- Administrator (*n* = 1) - Animal care (*n* = 1) - Council Planning Officer (*n* = 1) - Finance consultant (*n* = 1) - Housing officer (*n* = 1) - IT (*n* = 2) - Journalist (*n* = 1) - Higher education (*n* = 1) - Nurse (*n* = 1) - Photographer (*n* = 1) - Probation Officer (*n* = 1) - Student (*n* = 2) - Surveyor (*n* = 2) - Taxi driver (*n* = 1)
Employment status following injury	Returned to work (*n* = 12) Returned to education (*n* = 2) Not returned to work (*n* = 3)
Vocational rehabilitation	Received VR (*n* = 3); Did not receive VR (*n* = 14)

**Table 2:** Study 2 themes and subthemes.

Theme	Subthemes
1. Physical and psychological recovery	1.1. Physical recovery
	1.2. Psychological recovery
	1.3. Purposeful life engagement as part of recovery
2. Understanding of normality and managing expectations	2.1. Returning to normal
	2.2. Recovery expectations
3. Managing work and employer expectations	3.1. Managing the survivors’ return to work expectations
	3.2. Workplace understanding impact of injury, including invisible impacts
	3.3. Successfully managing expectations

## Data Availability

The data that participants have consented to share, will become available to potential researchers at the end of the study. Requests detailing the research aims and use of the data should be sent to the research team via email: ROWTATE@nottingham.ac.uk.
